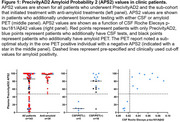# Determining eligibility for anti‐amyloid treatments using blood biomarkers

**DOI:** 10.1002/alz70856_097511

**Published:** 2025-12-24

**Authors:** Suzanne E. Schindler, Anna Hofmann, Madeline Paczynski, Zachary J Posey, Melissa Aldinger, Tammie L.S. Benzinger, John C. Morris, Barbara Joy Snider

**Affiliations:** ^1^ Washington University in St. Louis, St. Louis, MO, USA; ^2^ Washington University School of Medicine, St. Louis, USA; ^3^ Washington University School of Medicine, St. Louis, MO, USA; ^4^ Washington University School of Medicine, Saint Louis, MO, USA; ^5^ Washington University, Saint Louis, MO, USA

## Abstract

**Background:**

The Washington University Memory Diagnostic Center (MDC) in St. Louis, Missouri, sees approximately 4,000 patients with memory and thinking concerns annually and has been using blood biomarkers to assess for Alzheimer disease (AD) pathology and to determine eligibility for anti‐amyloid treatments.

**Methods:**

The MDC used the Roche Elecsys *p*‐tau181/Aβ42 CSF test and clinical visual read of Florbetaben or Florbetapir amyloid PET scans. For blood biomarker testing, the MDC used the C2N Diagnostics PrecivityAD2 blood test that incorporates the ratio of phosphorylated to non‐phosphorylated tau at position 217 and Aβ42/Aβ40 to generate a modeled value from 0 to 100 termed the amyloid probability score 2 (APS2). A positive APS2 value is >47.5. Clinical and demographic data were retrospectively collected from electronic health records.

**Results:**

A total of 143 patients with cognitive concerns were included who underwent a PrecivityAD2 test as part of their clinical evaluation. The average patient age was 74.5 ± 6.6 years (mean ± standard deviation), 48% were female, 94% were White, and 76% were positive by PrecivityAD2. A sub‐cohort of 34 patients were treated with the anti‐amyloid antibody lecanemab. The median APS2 value in the treated sub‐cohort was 97, with 75% of patients having an APS2 of 87 or higher. Twenty‐one patients additionally underwent either amyloid PET or CSF testing (Figure 1). PrecivityAD2 agreed with CSF tests in all fourteen patients with both tests (nine positive and 5 negative). PrecivityAD2 agreed with amyloid PET in six PET positive patients. PrecivityAD2 was negative in one PET positive patient, but the PET report noted, “Suboptimal study due to low signal‐to‐noise ratio,” suggesting the PET read may be false positive. If CSF or PET is considered the reference standard, PrecivityAD2 had a positive predictive value of 100% and negative predictive value of 83%.

**Conclusions:**

In 21 patients who underwent a second test with CSF or PET, all 15 patients who were positive by PrecivityAD2 were also positive by CSF or PET. Although this study is very small, these data support providing anti‐amyloid treatments based on high accuracy blood biomarker tests to patients with a high clinical likelihood of amyloid pathology.